# Prenatal Depression in Women in the Third Trimester: Prevalence, Predictive Factors, and Relationship With Maternal-Fetal Attachment

**DOI:** 10.3389/fpubh.2020.602005

**Published:** 2021-01-26

**Authors:** Ling Zhang, Lei Wang, Shu Cui, Qiuyu Yuan, Cui Huang, Xiaoqin Zhou

**Affiliations:** ^1^College of Mental Health and Psychological Science, Anhui Medical University, Hefei, China; ^2^Chaohu Hospital, Anhui Medical University, Hefei, China

**Keywords:** prenatal depression, predictive factors, the third trimester, pregnant women, maternal-fetal attachment intensity, maternal-fetal attachment quality

## Abstract

**Objective:** The prevalence of prenatal depression in pregnant women has found to be high, which may adversely affect the intimacy of a mother to her fetus. Few studies have investigated the relationship between prenatal depression and maternal-fetal attachment in pregnant Chinese women. This study is thus designed to evaluate the prevalence rate, predictive factors of prenatal depression in Chinese pregnant women in the third trimester of pregnancy, and the effect of prenatal depression on maternal-fetal attachment.

**Methods:** A total of 340 pregnant women in the third trimester of pregnancy were recruited from a hospital in Anhui Province. The Edinburgh Postpartum Depression Scale (EPDS) was rated to assess the prenatal depression; the Pittsburgh Sleep Quality Index (PSQI) and Zung Self-Rating Anxiety Scale (SAS) were used to assess sleep quality and anxiety level for all participants. The Maternal Antenatal Attachment Scale (MAAS) was used to assess maternal-fetal attachment.

**Results:** The prevalence of prenatal depression in the participants was high (19.1%) in our study. The scores of prenatal anxiety and sleep disorders were higher with prenatal depression than in those without prenatal depression (47.6 ± 9.5 vs. 38.9 ± 6.9; 8.3 ± 3.3 vs. 6.1 ± 2.7, all *p* < 0.01). MAAS quality was lower in prenatal depression women than those in non-prenatal depression women (43.8 ± 5.6 vs. 46.4 ± 4.5, *p* < 0.01). Correlation analysis showed that prenatal depression was associated with parity, prenatal education, education level, marital satisfaction, anxiety and sleep disorders (all *p* < 0.05). Furthermore, binary logistic regression results showed that anxiety and sleep disorders were risk factors for prenatal depression. Prenatal education, higher marriage satisfaction were protective factors for prenatal depression. In addition, correlation analysis also showed that prenatal depression was positively correlated with MAAS intensity, but negatively correlated with MAAS quality.

**Conclusions:** Our results indicated a high prevalence of prenatal depression in women in the third trimester. Prenatal education and higher marital satisfaction were protective factors for prenatal depression; antenatal anxiety and sleep disorders during pregnancy were risk factors for prenatal depression. Prenatal depression was negatively correlated with MAAS quality, but positively correlated with MAAS intensity.

## Introduction

Pregnancy is a challenging experience for women, both physically and psychologically, as it is usually considered to be stressful for them (1). In many developing countries, prenatal check-ups mainly focus on the physical health of pregnant women, with less focus on emotional and mental health issues. Among mental health problems of pregnant women, prenatal depression has come to the spotlight in recent years. Prenatal depression, a kind of emotional disorder that occurs during pregnancy, is mainly manifested through a bad mood, low interest and other low moods with a prevalence ranging from 10 to 29.6% (2, 3). The prevalence of prenatal depression is even higher in some developing countries (4). Prenatal depression may increase suicide rate during pregnancy and postnatal depressive disorders (5, 6). Moreover, depression during pregnancy was correlated with the neurodevelopmental disorders of fetus after birth and adverse obstetrical and neonatal outcomes; it also increased the incidence of mental health problems in children, later in their life (7, 8).

Furthermore, women with prenatal depression may be less sensitive to their fetus, thus affecting the intimate relationship between a mother and fetus (9, 10). It has been found that prenatal depression is also a risk factor for a poor mother-infant bonding (11). A previous study has reported that women with prenatal depression may have poorer MFA (12).

MFA was first put forward by Cranely, refers to the emotional connection between women and their unborn fetus, including a mother's thoughts, behavior, emotion and attitude toward the fetus (13). It could predict the occurrence of postpartum depression (14). High quality of MFA can promote the role recognition of pregnant women, facilitate the growth and development environment of the fetus in the uterus, and can ease postpartum anxiety and depression (15–17). Meanwhile, poor quality of MFA was related to low-quality of maternal-infant bond and less optimal early childhood development (18, 19).

There are few studies on the relationship between prenatal depression and MFA in China, especially in the third trimester, when MFA gradually become stable (13, 20). This study mainly investigated the prevalence rate and predictive factors of prenatal depression in Chinese pregnant women and the relationship between prenatal depression and MFA. We hypothesized that: 1) the prevalence rate of prenatal depression in the third trimester is high, and some demographic variables such as planned pregnancy, marital satisfaction are predictive factors; 2) prenatal depression affects the expression of maternal attachment to the fetus.

## Methods

### Participants and Procedure

Pregnant women who underwent prenatal examination from September 2019 to November 2019 at Chaohu Hospital of Anhui Medical University were selected as the research participants. The hospital is a tertiary general hospital affiliated to a provincial university. The inclusion criteria for pregnant women were as follows: 1) pregnant women who were in the third trimester; 2) were aged between 18 and 40 years; 3) women with singleton pregnancy. However, those who met the following criteria were excluded from the study: 1) participants with a previous history of mental disorders; 2) pregnant women with a high-risk pregnancy; 3) pregnant women with a previous history of miscarriage.

Convenience sampling was used in this study. Unified training and standardized instructions were given to all pregnant women. After excluding all incomplete and invalid questionnaires, 340 pregnant women were included in this study. All the women signed informed consent forms before participating. The ethics committee of Chaohu Hospital of Anhui Medical University approved the research protocol.

### Instruments

#### Self-Made Demographic Questionnaire

We used a self-made demographic questionnaire to evaluate contents like age, gestational weeks, education, parity, planned pregnancy, body shape change, prenatal education, work status, marriage satisfaction of the participants. Except for age, gestational weeks, education level and parity, other demographic data were inquired about by following standardized questions: “Did you plan to get pregnant?”; “Can you accept body shape change due to pregnancy?”; “Have you ever participated in a standardized prenatal education course?”; “Did you keep working during the pregnancy?”; “Are you satisfied with your marriage?” All questions were to be answered by choosing either “yes” or “no.”

#### Prenatal Depression

In this study, the Edinburgh Postnatal Depression Scale (EPDS) was used to evaluate the prenatal depression (21). It includes 10 items, which were to be rated from zero (no symptoms) to three (severe symptoms), the final score was from 0 to 30 points. A higher score indicates more severe depressive symptoms. We regarded a total EPDS score ≥12, indicating prenatal depression. EPDS scale has been validated in different cultures including China, and the cut-off value of 12 has also been proven and practiced frequently (22–25). The content validity ratio is 0.93.

#### Prenatal Sleep Quality

The Pittsburgh Sleep Quality Index (PSQI) was used to assess sleep during pregnancy. It includes sleep quality, sleep latency, sleep duration, sleep efficiency, sleep disturbances, sleep medication, and daytime dysfunction (26). The sum of these seven indicators is an overall measure of sleep quality, where higher scores mean worse sleep quality. People with a total score of PSQI ≥ 5 were identified as having sleep disorders.

#### Prenatal Anxiety

The Zung Self-Rating Anxiety Scale (SAS) was used to assess prenatal anxiety. This scale includes 20 items, and is divided into four grades; the scores of 20 items add up to a rough score (27). The standard score is obtained by multiplying the total score by 1.25 and the standard threshold of 50 is often used to diagnose anxiety. In this study, the higher scale score demonstrated more severe anxiety symptoms.

#### MFA

We used the Maternal Antenatal Attachment Scale (MAAS) to evaluate the MFA of the participants (28). MAAS is a self-rating scale consisting of 19 questions with a 5-point scoring system. According to the results of score, it can be divided into two dimensions: MAAS quality and MAAS intensity. The quality of MAAS indicates the emotional experience related to the fetus, while the intensity of MAAS indicates the time and effort that the pregnant woman spends on the fetus.

### Data Analysis

The Statistical Package for Social Sciences 23.0 (SPSS23.0) was used to analyze the data, and the normal distribution of continuous variables was tested. We compared the differences in demographics and mental health variables between the depression group and the non-depression group. The continuous variables used the *t*-test or the Mann-Whitney U test depending on whether the distribution was normal; the chi-square test was used to for categorical variables. Furthermore, Spearman's correlation was performed to examine the correlations between depression score, demographic data and mental health variables as well as the correlations between MAAS intensity or MAAS quality and depression score. Finally, after controlling confounding factors, we used a binary regression analysis to identify significant predictive variables associated with prenatal depression. A two-tailed *p*-value of 0.05 was considered to be statistically significant.

## Results

### Demographic Characteristics and Mental Health Variables of the Participants

A total of 340 participants were included in this research. Among them, there was a 19.1% (65/340) prevalence of prenatal depression in our study. The average age of participants was 28.3[standard deviation (SD) = 4.4] years and the gestational week was 35.4 (SD = 2.7) weeks. Approximately, half of all participants had high school or junior college education (51.8%), education less than high school and those with bachelor's or higher accounted for 22.3 and 25.9%, respectively. Moreover, 61.8% of the participants were nulliparous women. The majority of women had a planned pregnancy (61.8%). Most pregnant women reported that they could accept body shape change during pregnancy (80.9%). Of the 340 pregnant women in the third trimester, the majority of participants had prenatal education during pregnancy (65.3%) and 41.8% of pregnant women were employed. Of the total participants, 82.1% of the participants were satisfied with their marriage. Furthermore, the proportions of those suffering from anxiety and sleep disorders among the participants were 14.1% and 69.1%, respectively.

### Comparison of Demographics, Mental Health Variables, and MFA Between Women With Prenatal Depression and Women With Non-prenatal Depression

As shown in Table 1, differences in prenatal education and marriage satisfaction between women with prenatal depression and women with non-prenatal depression were statistically significant (all *p* < 0.05). In addition, the score of prenatal anxiety and sleep disorders both were higher in women with prenatal depression than those with no prenatal depression (47.6 ± 9.5 vs. 38.9 ± 6.9; 8.3 ± 3.3 vs. 6.1 ± 2.7, all *p* < 0.01). However, there were no significant differences in age, gestational weeks, education level, parity, planned pregnancy, body shape change and work status between the two groups (all *p* > 0.05). Moreover, pregnant women with prenatal depression had higher lower MAAS quality (43.8 ± 5.6 vs. 46.4 ± 4.5, *p* < 0.01). However, there was no significant difference in the total score of MAAS and MAAS intensity was noted.

**Table 1:** Comparison of demographics, mental health variables and MFA between women with prenatal depression and women with non-prenatal depression.

	**All participants**	**Prenatal depression**	**Non-prenatal depression**	**t/Z/X^2^**	***P***
	**(*n =* 340)**	**(*n =* 65; 19.1%)**	**(*n =* 275;80.9%)**		
Age (years)	28.3 ± 4.4	28.2 ± 4.5	28.3 ± 4.4	−0.118	0.906
Gestational weeks	35.4 ± 2.7	35.9 ± 2.4	35.3 ± 2.7	−1.106	0.269
Less than high school	76 (22.3%)	9 (13.8%)	67 (24.4%)	3.547	0.170
High school or junior college	176 (51.8%)	36 (55.4%)	140 (50.9%)		
Bachelor degree or higher	88 (25.9%)	20 (30.8%)	68 (24.7%)		
Nulliparous women	210 (61.8%)	43 (66.2%)	167 (60.7%)	0.656	0.418
Planned pregnancy	210 (61.8%)	43 (66.2%)	167 (60.7%)	0.656	0.418
Can accept body change	275 (80.9%)	54 (60.7%)	221 (80.4%)	0.250	0.617
Prenatal education	222 (65.3%)	28 (43.1%)	194 (43.1%)	4.183	0.041
Employed	142 (41.8%)	26 (40.0%)	116 (42.2%)	0.103	0.748
Marital satisfaction	279 (67.7%)	44 (15.8%)	235 (85.5%)	11.266	0.001
Prenatal anxiety	48 (14.1%)	37 (56.9%)	11 (4.0%)	44.402	<0.01
Anxiety score	40.6 ± 8.2	47.6 ± 9.5	38.9 ± 6.9	−6.931	<0.01
Sleep disorders	235 (69.1%)	57 (87.7%)	178 (64.7%)	12.990	<0.01
Total score of PSQI	6.5 ± 3.0	8.3 ± 3.3	6.1 ± 2.7	−4.821	<0.01
Total score of MAAS	73.4 ± 7.3	72.4 ± 7.3	73.6 ± 7.3	0.225	0.240
MAAS intensity	27.5 ± 4.7	28.4 ± 5.0	27.3 ± 4.6	−1.756	0.079
MAAS quality	45.9 ± 4.8	43.8 ± 5.6	46.4 ± 4.5	−3.524	<0.01

*PSQI, Pittsburgh Sleep Quality Index; MAAS, Maternal Antenatal Attachment Scale*.

### Correlation of Prenatal Depression, Demographic, and Mental Health Variables

Spearman's correlation analysis showed that depression of pregnant women was correlated with parity (r = −0.111, *p* = 0.041), prenatal education (r = 0.179, *p* = 0.001), education level (r = 0.154, 0.04), marital satisfaction (r = 0.167, *p* = 0.002), anxiety (r = 0.578, *p* < 0.001), and sleep disorders (r = 0.348, *p* < 0.001). In addition, binary logistic regression was performed on all demographics and mental health variables. The four statistically significant variables to predict prenatal depression were prenatal education, marital satisfaction, anxiety, and sleep disorders. The coefficients of these variables were presented in Table 2. Women who had prenatal education [odds ratio (OR) 0.261, 95% CI 0.135, 0.506] and those with higher marital satisfaction (OR 0.331, 95% CI 0.162, 0.1677) were less likely to develop prenatal depression. Women who experienced prenatal anxiety (OR 6.405, 95% CI 2.998, 13.681) and sleep disorders during pregnancy (OR 2.547, 95% CI 1.087, 6.968) were at a higher risk of suffering from prenatal depression.

**Table 2:** Predictors generated by Binary Logistic Regression with depression as the dependent variables.

	***P***	**OR**	**95% CI**	
			**Lower**	**Upper**
Prenatal education	<0.001	0.261	0.135	0.506
Marital satisfaction	0.002	0.331	0.162	0.677
Prenatal anxiety	<0.001	6.405	2.998	13.681
Sleep disorders	0.031	2.547	1.087	5.968

### Correlation Between Prenatal Depression Score and MFA Dimension

**Figure 1:**
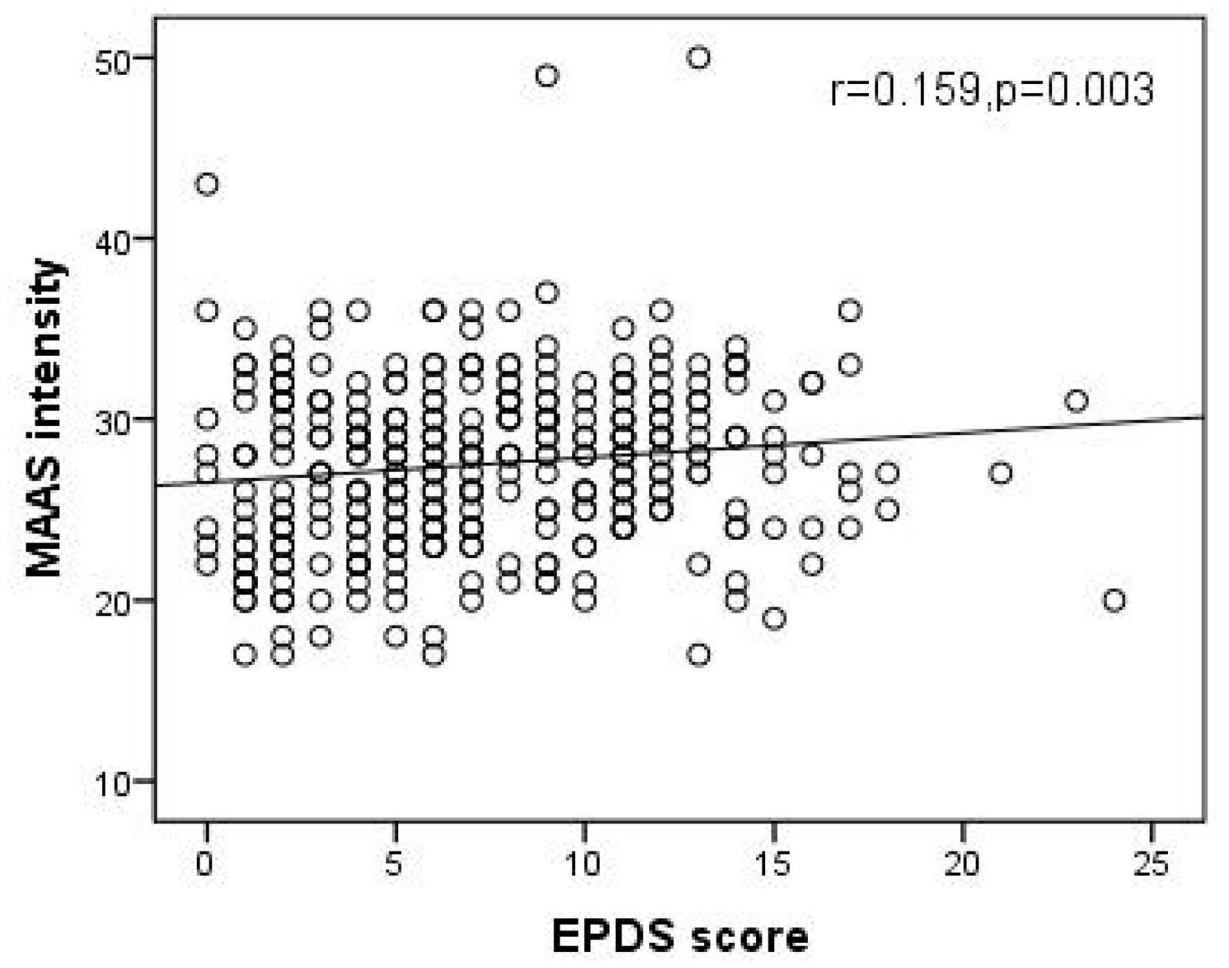
Significant positive correlation of EPDS score with MAAS intensity. (Spearman's r = 0.159, p = 0.003). EPDS, Edinburgh postpartum depression scale; MAAS, Maternal Antenatal Attachment Scale.

**Figure 2:**
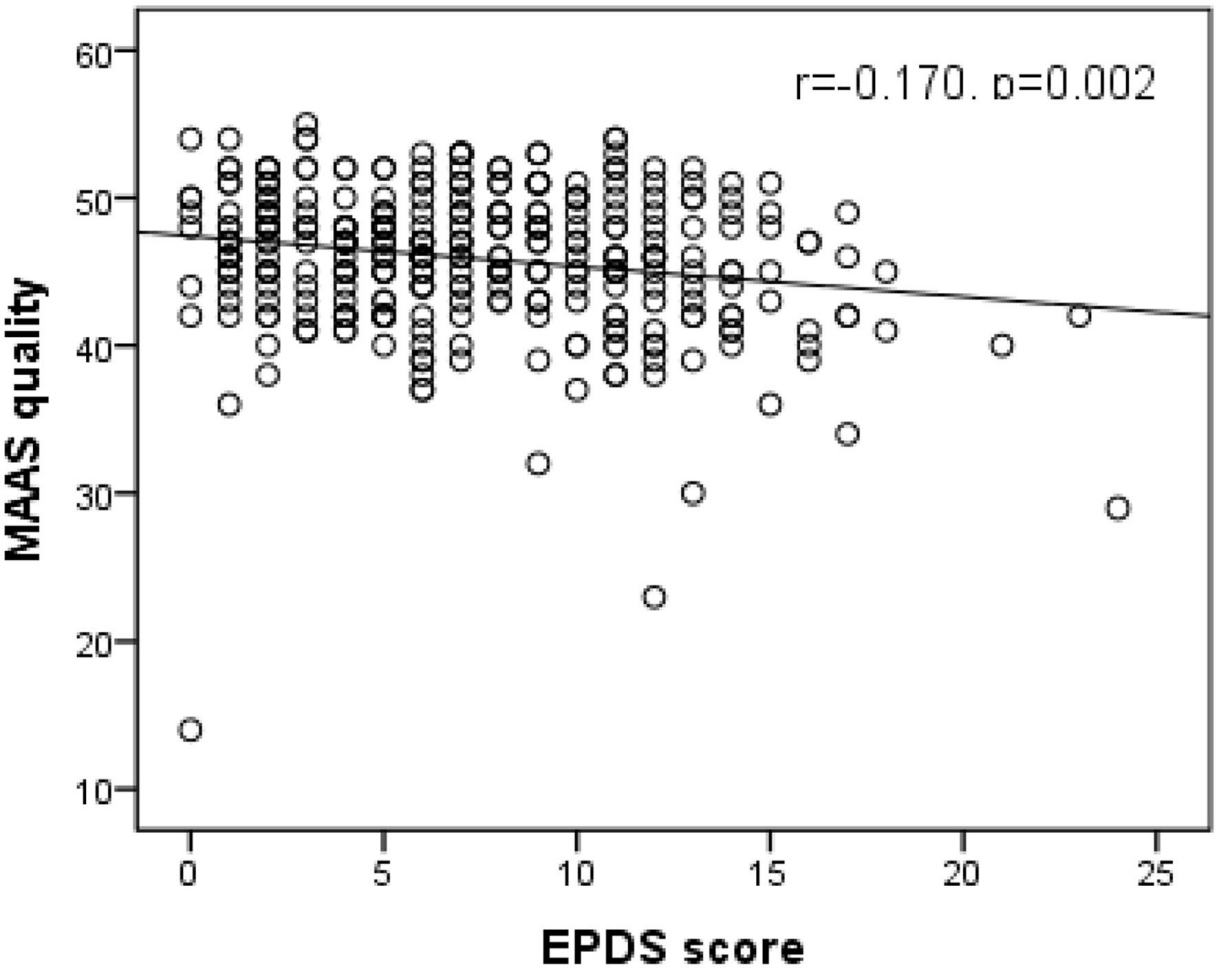
Significant negative correlation of EPDS score with MAAS quality. (Spearman's r = −0.170, p = 0.002). EPDS, Edinburgh postpartum depression scale; MAAS, Maternal Antenatal Attachment Scale.

As shown in Figures 1, 2, Spearman's correlation analysis showed that prenatal depression was positively correlated with MAAS intensity but negatively correlated with MAAS quality (r = 0.159, *p* = 0.003; r= −0.170, *p* = 0.002).

## Discussion

There are three main findings from this study. First, the prevalence of depression was high in the third trimester. Second, some variables, including antenatal anxiety and sleep disorders were risk factors for prenatal depression, while prenatal education and higher marriage satisfaction were the protective factors. Third, prenatal depression was positively correlated with MAAS intensity and negatively correlated with MAAS quality.

The results showed that the prevalence of prenatal depression was 19.1%, which was higher than that reported in an existing literature (29). As all participants were in the third trimester of pregnancy, and nearing the date of delivery, which might lead to changes in their social roles, they were more worried about the upcoming delivery and the health of their fetus. Thus, the high prevalence of prenatal depression has become an important public health problem, which should be focused on and appropriate intervention should be taken.

The first risk factor for prenatal depression was antenatal anxiety, which was similar to previous studies (30, 31). In this study, approximately 14.1% of pregnant women had anxiety symptoms during their pregnancy. Li also found that the level of anxiety had a direct that the level of anxiety had direct effect on prenatal depression (32). Moreover, sleep disorders were another risk factor for prenatal depression. Prenatal depression and sleep disorders can also be inter-related. Due to physical discomfort and heavy physical burden in the period of late pregnancy, difficulties in breathing during sleep and maintaining a normal flat sleeping posture crops up. Therefore, sleep disorders are a kind of common symptom in the third trimester of pregnancy. This may also be an important reason for the high prevalence (69.1%) of sleep disorders in this study. It also reminds us that in the intervention of prenatal depression, anxiety and sleep disorders of pregnant women must be considered.

This study found that pregnant women who received prenatal education during pregnancy were less likely to have symptoms of prenatal depression. Education during pregnancy not only reduced complaints of pregnancy, but also promoted the quality of pregnancy (33). Furthermore, women with prenatal education could better adapt to the changes during pregnancy (34). In addition, higher marriage satisfaction was found to be a protective factor for prenatal depression. This finding was consistent with a previous research (24). Perhaps, in a bad marriage, the spouse cannot play a supportive role to ameliorate the mood (35). Due to the lack of support and communication from their husbands, pregnant women in a bad marriage were more vulnerable to prenatal depression. A Korean study also pointed out that it is necessary to improve marriage satisfaction counseling programs to prevent pregnant women from suffering from depression while transitioning to motherhood (36).

It was found that pregnant women in the prenatal depression group had lower MAAS quality. Another result of this study showed that prenatal depression was negatively correlated with MAAS quality. These results were similar to previous studies (37, 38). One explanation could be that by Alhusen et al., who proposed that prenatal depression could affect response of pregnant women to pregnancy, and lead to a negative impact on MFA (39). Moreover, there was no statistically significant difference in MAAS intensity between pregnant women in the depression group and the normal group. The correlation analysis showed that depression scores were positively correlated with MAAS intensity. This positive correlation was also reflected in a systematic review, which showed that prenatal depression was positively correlated with some dimensions of antenatal attachment scale, such as “physical contact with the fetus, empathy, fantasy and the maternal sensitivity to the fetus” (40–42). One possible explanation was that positive correlation between prenatal depression and MAAS intensity may not mean that there was a closer relationship between a mother and fetus. Attention to fetus of pregnant women with higher prenatal depression scores was not necessarily related to positive effects (40). In addition, pregnant women with higher depression were more likely to be affected by body perception and feelings such as fetal movement and other physical changes caused by pregnancy, which may not be considered positive (43). Furthermore, pregnant women with higher depression would treat them as a negative physical indicator due to negative cognition. Another possible explanation is that women with higher prenatal depression would be more worried that depression may affect the fetus. However, because of the influence of prenatal depression, their efficiency of communication with the fetus and their ability to develop a close relationship with it would be hampered, which led to low MAAS quality. Therefore, the relationship between maternal depression and MAAS intensity requires further study. The results of the relationship between prenatal depression and MFA in this study provide some new perspectives for subsequent interventions and to help expectant mothers make a better transition to the role of motherhood.

There are, however, some limitations in our study. The first limitation is that the study has a cross-sectional design. Postpartum depression is not tracked, which should be analyzed and discussed in our future research. Moreover, using self-reported questionnaires may result in underreporting of sensitive or stigmatizing behaviors such as depression. It is also to be noted that self-selection bias cannot be avoided. Another potential limitation of the study is that the sample size is a bit small, but there were differences in variables between the depression group and the normal group of pregnant women. We shall carry out a larger sample study in future studies. Despite these limitations, this study explored the high prevalence of prenatal depression in women in the third trimester of pregnancy as well as predictive factors for prenatal depression. It may provide some insights into the prevention of prenatal depression and pregnancy health care. Additionally, the impact of prenatal depression on different dimensions of MFA also provides us with significant implications of the relationship between prenatal depression and MFA. According to a study conducted in China in 2016, there were only 1.7 psychiatrists and 0.05 psychiatric hospitals per 100,000 people, and even fewer institutions that conduct mental health screening and intervention for pregnant women (44). Therefore, the government and social policy makers should develop and integrate a sound health system, increase the proportion of mental health screening of pregnant women in prenatal examination, and thus improve the current situation. It should also promote the mental health of pregnant women and improve their pregnancy outcome.

## Data Availability Statement

The original contributions presented in the study are included in the article/supplementary material, further inquiries can be directed to the corresponding author/s.

## Ethics Statement

The studies involving human participants were reviewed and approved by The ethics committee of Chaohu Hospital of Anhui Medical University. The patients/participants provided their written informed consent to participate in this study.

## Author Contributions

The manuscript was designed and written by authors LZ and LW. Data was collected by LZ and SC, analyzed by LZ, QY, SC, CH, and verified by XZ. All authors read and agreed to the final manuscript.

## Conflict of Interest

The authors declare that the research was conducted in the absence of any commercial or financial relationships that could be construed as a potential conflict of interest.

## Abbreviations

EPDS: Edinburgh Postpartum Depression Scale
PSQI: Pittsburgh Sleep Quality Index
SAS: Self-Rating Anxiety Scale
MAAS: Maternal Antenatal Attachment Scale
MFA: Maternal-fetal attachment
CI: Confidence interval.
